# Great American Smokeout — November 21, 2013

**Published:** 2013-11-15

The Great American Smokeout, sponsored by the American Cancer Society, is an annual event that encourages smokers to make a plan to quit, or to plan in advance and quit smoking on that day, in an effort to stop permanently (1). This year, the Smokeout will be held on November 21.

Fifty years after the release of the first Surgeon General’s report on smoking and health, remarkable progress has been made. Since 1964, smoking prevalence among U.S. adults has been reduced by half. Unfortunately, tobacco use remains the leading preventable cause of disease, disability, and death in the United States (2).

In 2010, nearly two out of three adult smokers wanted to quit, and more than half had made a quit attempt for >1 day in the preceding year (3). However, an estimated one out of five U.S. adults still smokes (2).

Quitting smoking is beneficial to health at any age and has immediate and long-term benefits. Getting help through counseling or medications can double or triple the chances of quitting successfully (4).

Additional information and support for quitting is available by telephone (800-QUIT-NOW [800-784-8669]). Additional quit support and real stories of persons who have quit successfully are available on CDC’s Tips from Former Smokers website at http://www.cdc.gov/tips.
